# Hypertension and Its Determinants Among White‐Collar Workers: A Cross‐Sectional Study Focusing on Lifestyle Risk Factors and Health Literacy

**DOI:** 10.1155/bmri/3545756

**Published:** 2026-01-08

**Authors:** Sandeepa Karki, Shyam Sundar Budhathoki, Shishir Paudel, Dhurba Khatri, Anup Ghimire, Deepak Kumar Yadav, Paras Kumar Pokharel

**Affiliations:** ^1^ Kathmandu Institute of Child Health, Kathmandu, Nepal; ^2^ Department of Primary Care and Public Health, School of Public Health, Imperial College London, London, UK, imperial.ac.uk; ^3^ Faculty of Science, Health and Technology, Nepal Open University, Lalitpur, Nepal; ^4^ School of Public Health and Community Medicine, B.P. Koirala Institute of Health Sciences, Dharan, Nepal, bpkihs.edu

## Abstract

**Background:**

Hypertension is a growing public health concern, particularly among white‐collar workers exposed to sedentary lifestyles and occupational stress. This study examines the prevalence and determinants of hypertension among commercial bank employees in Sunsari, Nepal, with a focus on lifestyle risk factors and health literacy.

**Methods:**

A cross‐sectional study was conducted among 240 bank employees using stratified random sampling. Data were collected through a self‐administered questionnaire adapted from the WHO STEPS survey and clinical measurements of blood pressure, BMI, and waist circumference. Hypertension was classified based on the JNC 8 guidelines. Descriptive statistics, chi‐square tests, and independent *t* tests were used for preliminary analysis. Logistic regression was performed to identify independent predictors of hypertension, controlling for potential confounders.

**Results:**

The prevalence of hypertension was 62.1% (95% CI 58.6–65.6), with 47.5% in Stage I and 14.6% in Stage II. Males had significantly higher odds of hypertension than females (aOR 2.237, 95% CI 1.170–4.276). Behavioral risk factors such as alcohol consumption (aOR 4.732, 95% CI 1.386–16.160) and frequent processed food intake (aOR 2.640, 95% CI 1.024–7.096) were significantly associated with hypertension. Overweight (aOR 1.819) and obesity (aOR 1.575) were also found to be the major risk factors. Lower health literacy scores, particularly in healthcare engagement and self‐management, were associated with hypertension.

**Conclusion:**

The high prevalence of hypertension among bank employees highlights the need for workplace interventions promoting healthy lifestyles, routine screenings, and health literacy programs to improve hypertension awareness and management.

## 1. Introduction

Hypertension, often referred to as a “silent killer,” is a leading global public health concern, contributing significantly to the burden of non‐communicable diseases (NCDs) [1, 2]. Hypertension is a major risk factor for cardiovascular diseases, accounting for 54% of strokes and 47% of coronary diseases globally [3]. The global prevalence of hypertension is rising, particularly in low‐ and middle‐income countries (LMICs), where nearly two‐thirds of hypertensive individuals reside [4]. Evidence from the multinational study on Global Aging and Adult Health (SAGE) revealed high prevalence of hypertension in all SAGE countries namely China, Ghana, India, Mexico, Russian Federation, and South Africa disclosed a high prevalence of hypertension in developing nations [5, 6]. Undiagnosed and uncontrolled hypertension could lead to severe consequences such as ischemic heart disease, stroke, kidney disease, dementia, and premature mortality [7, 8].

Nepal, like many developing nations, is undergoing an epidemiological transition, where non‐NCDs such as hypertension are rising alongside infectious diseases [9, 10]. The 2019 WHO STEPS survey estimated that 24.5% of Nepalese adults suffer from hypertension [11]. A systematic review and meta‐analysis reported a pooled prevalence of hypertension in Nepal at 28.4% (95% CI 22.4–34.7%) [10]. This increasing burden is linked to urbanization, dietary shifts, sedentary lifestyles, and occupational stress. In response, aligning with global efforts, Nepal has implemented a Multisectoral Action Plan for the Prevention and Control of Non‐Communicable Diseases (2014–2020) to mitigate this rising burden [12].

Workplace environments significantly influence hypertension risk due to sedentary behavior, job‐related stress, and unhealthy lifestyle factors. Globally, studies have shown that financial institution workers, a subset of white‐collar professionals exhibit a notable prevalence of hypertension due to sedentary behavior, work‐related stress, limited physical activity, and unhealthy lifestyle practices [13, 14]. Studies from South Asia reveal a high prevalence of hypertension among bank employees, ranging from 24.4% in Bangladesh to 44.3%–69.5% in India [14–16]. However, despite the growing concern about hypertension in working populations in neighboring countries, hypertension in occupational settings particularly among white‐collar workers in Nepal remains underexplored.

Additionally, health literacy which is a crucial determinant of overall health and wellbeing as well as hypertension related awareness, prevention, and management remains poorly understood in Nepalese occupational settings. Evidence suggests that individuals with low health literacy often fail to recognize early symptoms, adhere to treatment, or engage in preventive behaviors [17–19]. This study assesses the prevalence of hypertension and its associated factors among financial institution employees in Sunsari, Nepal. Additionally, it examines the level of health literacy among white‐collar workers in financial institutions. Findings from this study will contribute to workplace health promotion strategies, targeted hypertension prevention programs, and national NCD control efforts in Nepal.

## 2. Method

### 2.1. Study Design and Study Setting

This cross‐sectional study was conducted among commercial bank employees in Sunsari District, Nepal. Sunsari consists of two sub‐metropolitan cities and four municipalities, with several commercial banks operating in these regions. The study focused on permanent administrative and managerial employees working in these institutions.

### 2.2. Sample Size Determination

The sample size was calculated using Cochran’s formula for estimating a proportion (*n* = *z* ^2^ *p* *q*/*d* ^2^). The Nepal Demographic and Health Survey (NDHS) 2016 reported 19.9% study participants were diagnosed as hypertensive [20]. Using a 5% allowable error and a 95% confidence interval, the required sample size was determined to be 245. Of the 245 participants approached, 240 provided complete responses.

A stratified random sampling approach was employed to ensure adequate representation of bank employees across different administrative regions. First, all commercial banks in Sunsari District were listed and stratified according to location, distinguishing between sub‐metropolitan cities and municipalities. Two of the four municipalities were then randomly selected using a lottery method. Within each selected area, banks were chosen randomly, with proportional allocation based on the total number of banks in each region. From each selected institution, a list of eligible employees was obtained. Eligibility criteria included permanent employment in an administrative or managerial role and willingness to provide informed consent. Employees on temporary contracts, security staff, and those unavailable after three contact attempts were excluded. Participants were selected from the eligible pool using a random number generator, with eight employees per bank included in the final sample.

### 2.3. Data Collection

Data collection was conducted during the months of March 2020 using a self‐administered questionnaire and clinical measurements. The questionnaire was adapted from the WHO STEPS survey instrument to ensure validity and relevance to the study objectives. The questionnaire comprised three sections: sociodemographic details, behavioral risk factors, and health literacy. Sociodemographic details included age, gender, marital status, education, and job role. Behavioral risk factors assessed tobacco and alcohol use, dietary habits, physical activity, and stress levels. Health literacy was evaluated using a validated Health Literacy Questionnaire (HLQ) [21] designed to assess different domains of health literacy, which has been validated in Nepal [22].

In addition to the questionnaire, clinical measurements were recorded. Blood pressure was measured three times at 5‐min intervals using a validated sphygmomanometer and stethoscope by a medical officer. The average systolic blood pressure (SBP) and diastolic blood pressure (DBP) values were used for analysis, with hypertension categorized according to the Joint National Committee (JNC 8) guidelines [23]. Anthropometric data, including height, weight, and waist circumference, were measured using standardized techniques. The body mass index (BMI) was calculated and classified using WHO BMI criteria. To ensure privacy and minimize potential bias, all measurements were conducted in a designated area within the workplace.

### 2.4. Variables

The outcome variable of interest was hypertension, classified based on the Joint National Committee (JNC 8) guidelines [23]. Hypertension status was categorized into normal (systolic blood pressure [SBP] of < 120 mmHg and a diastolic blood pressure [DBP] of < 80 mmHg), elevated blood pressure (prehypertension) as SBP 120–139 mmHg or DBP 80–89 mmHg, Stage I hypertension as SBP 140–159 mmHg or DBP 90–99 mmHg, and Stage II hypertension as SBP ≥ 160 mmHg or DBP ≥ 100 mmHg. For analysis, the data were dichotomized into normal/elevated (normal + elevated categories) and hypertensive (Stage I + Stage II hypertension). Independent variables included sociodemographic, behavioral, and health‐related factors. Sociodemographic variables encompassed age, gender, marital status, education level, and job role. Behavioral variables included tobacco and alcohol consumption, dietary patterns, physical activity, and stress levels, with physical activity assessed using metabolic equivalent of task (MET) scores. Health‐related factors included family history of hypertension, diabetes, and other chronic conditions, as well as BMI and central obesity.

### 2.5. Statistical Analysis

The collected data were entered into Microsoft Excel 2016 using validation command to ensure data accuracy and consistency by detecting and correcting potential entry errors. The validated dataset was imported to Statistical Package for the Social Sciences Version 16. Descriptive statistics were used to summarize participants’ sociodemographic and clinical characteristics. Continuous variables were presented as means and standard deviations, while categorical variables were expressed as frequencies and percentages. The normality of the health literacy scale scores was checked before applying independent *t* tests to assess the relationship between health literacy scores in each domain and hypertension status. Pearson chi‐square tests was used to assess associations between categorical variables and hypertension status. Variables that were statistically significant in the bivariate analysis (*p* < 0.05) were included in a multivariable logistic regression model to identify independent predictors of hypertension. The variance inflation factor (VIF) was calculated to check for multicollinearity among independent variables, with a threshold of VIF > 5 considered indicative of multicollinearity concerns. Model fit was assessed using the Hosmer–Lemeshow test, and adjusted odds ratios (aORs) with 95% confidence intervals (CIs) were reported to estimate the strength of associations.

### 2.6. Ethical Considerations

Ethical clearance was obtained from the Institutional Review Committee of B.P. Koirala Institute of Health Sciences (Ref. code: IRC/1358/018). Written informed consent was obtained from all participants after providing a detailed explanation of the study’s objectives and procedures. Confidentiality and anonymity were maintained throughout the study, and participants were assured that their information would be used solely for research purposes.

## 3. Results

The prevalence of hypertension among participants was alarmingly high, with 62.1% (95% CI: 58.6–65.6) classified as hypertensive. Among these, 47.5% had Stage I hypertension, and 14.6% were in Stage II. Only 7.5% of participants reported a previous diagnosis of hypertension, highlighting a substantial gap in awareness and diagnosis. Meanwhile, 37.9% of participants had normal or elevated blood pressure levels, with elevated levels accounting for 23.8% of cases (Table 1).

**Table 1:** Prevalence of hypertension among employees.

	**n**	**% (95% CI for proportion)**
Blood pressure categories		
Normal	34	14.2 (11.7–16.7)
Elevated	57	23.8 (20.7–26.9)
Stage I hypertension	114	47.5 (43.9–51.1)
Stage II hypertension	35	14.6 (12.0–17.2)
Hypertension status		
Yes	149	62.1 (58.6–65.6)
No	91	37.9 (34.4–41.4)
Diagnosed history of hypertension		
Yes	18	7.5 (5.6–9.4)
No	222	92.5 (90.6–94.4)

The mean age of participants was approximately 30.57 ± 6.82, with the majority (52.1%) aged 20–29 years, followed by 38.8% aged 30–39 years, and 9.2% aged ≥ 40 years. Hypertension prevalence increased significantly with age (*p* = 0.010), peaking at 81.8% among participants aged ≥ 40 years. Males represented 64.2% of the sample, with hypertension prevalence significantly higher among males (70.8%) than females (46.5%; *p* < 0.001). Ethnicity was almost evenly distributed, with 50.4% of participants from privileged ethnic groups and 49.6% from underprivileged groups. However, hypertension prevalence did not significantly differ between these groups (*p* = 0.273). Regarding education, a majority of participants held graduate (45.0%) or postgraduate degrees (42.1%), while only 12.9% had secondary‐level education. Hypertension prevalence did not differ significantly across educational levels and their nature of work. Duration in the current job showed a significant association with hypertension (*p* = 0.047) (Table 2).

**Table 2:** Socio‐demographic profile of the participants.

**Characteristics**	**n** **(%)**	**Hypertension**		**Chi-square (*p* value)**
		**Presence** **n** **(%)**	**Absence** **n** **(%)**	
Age				
20–29 years	125 (52.1)	67 (53.6)	58 (46.4)	9.253 (0.010∗)
30–39 years	93 (38.8)	64 (68.8)	29 (31.2)	
≥ 40 years	22 (9.2)	18 (81.8)	4 (18.2)	
Gender				
Male	154 (64.2)	109 (70.8)	45 (29.2)	13.806 (< 0.001∗∗)
Female	86 (35.8)	40 (46.5)	46 (53.5)	
Ethnicity				
Privileged	121 (50.4)	78 (65.5)	41 (34.5)	1.202 (0.273)
Underprivileged	119 (49.6)	71 (58.7)	50 (41.3)	
Marital status				
Unmarried	102 (42.5)	62 (60.8)	40 (39.2)	0.127 (0.721)
Married	138 (57.5)	87 (63.0)	51 (37.0)	
Education				
Secondary	31 (12.9)	20 (64.5)	11 (35.5)	1.857 (0.395)
Graduate	108 (45.0)	62 (57.4)	46 (42.6)	
Postgraduate	101 (42.1)	67 (66.3)	34 (33.7)	
Nature of job				
Bank teller	84 (35.0)	52 (61.9)	32 (38.1)	3.353 (0.340)
Marketing representative/loan officer	70 (29.2)	39 (55.7)	31 (44.3)	
Internal auditor/data processing officer	61 (25.4)	39 (63.9)	22 (36.1)	
Bank manager	25 (10.4)	19 (76.0)	6 (24.0)	
Duration in job				
< 1 years	58 (24.2)	35 (60.3)	23 (39.7)	7.934 (0.047∗)
1–4 years	68 (28.3)	40 (59.8)	28 (41.2)	
5–9 years	63 (26.3)	34 (54.0)	29 (46.0)	
≥ 10 years	51 (21.3)	40 (78.4)	11 (21.6)	

^∗^Statistical significance at *p* < 0.05.

^∗∗^Statistical significance at *p* < 0.001.

Among the participants, 21.7% smoked tobacco, 7.9% consumed smokeless tobacco, and 9.6% reported alcohol consumption in the past 30 days. Alcohol consumption in the past 30 days was reported by 9.6% of participants, with a significantly higher prevalence of hypertension among drinkers (78.3%) compared to non‐drinkers (66.1%; *p* = 0.032). In terms of dietary habits, 60.4% of participants primarily used sunflower oil for cooking, while 33.8% used mustard oil. Hypertension prevalence was higher among sunflower oil users (66.2%) than mustard oil users (51.9%). Similarly, participants who consumed processed foods ≥ 7 times per week exhibiting the highest prevalence (73.9%), compared to those who consumed such foods rarely (47.4%). The majority of participants had a normal BMI (58.8%), followed by overweight (32.9%) and obese (8.3%). Hypertension prevalence increased with BMI, with obese individuals exhibiting the highest prevalence (80.0%; *p* = 0.013). Physical activity levels were predominantly low among participants (67.5%), but no significant association was found between physical activity and hypertension (Table 3).

**Table 3:** Lifestyle and behavioral factors related to hypertension.

**Characteristics**	**n** **(%)**	**Hypertension**		**Chi-square (*p* value)**
		**Presence** **n** **(%)**	**Absence** **n** **(%)**	
Smoking tobacco products				
Yes	52 (21.7)	36 (69.2)	16 (30.8)	1.441 (0.230)
No	188 (78.3)	113 (60.1)	75 (39.9)	
Consumption of smokeless tobacco				
Yes	19 (7.9)	14 (73.7)	5 (26.3)	1.180 (0.277)
No	221 (92.1)	135 (61.1)	86 (38.9)	
Exposure to passive smoking at home				
Yes	16 (6.7)	11 (68.8)	5 (31.3)	0.324 (0.569)
No	224 (93.3)	138 (61.6)	86 (38.4)	
Exposure to passive smoking at work				
Yes	31 (12.9)	20 (64.5)	11 (35.5)	0.090 (0.765)
No	209 (87.1)	129 (61.7)	80 (38.3)	
Alcohol consumption (past 30 days)				
Yes	23 (9.6)	18 (78.3)	5 (21.7)	6.907 (0.032∗)
No	124 (51.7)	82 (66.1)	42 (33.9)	
Never consume alcohol	93 (38.8)	49 (52.7)	44 (47.3)	
Cooking oil in household				
Mustard oil	81 (33.8)	42 (51.9)	39 (48.1)	6.266 (0.043∗)
Sunflower oil	145 (60.4)	96 (66.2)	49 (33.8)	
Other oil (plant based)	14 (5.8)	11 (78.6)	3 (21.4)	
Consumption of processed food				
Often (≥ 7 times a week)	69 (28.8)	51 (73.9)	18 (26.1)	7.808 (0.020∗)
Sometimes (3–6 times a week)	133 (55.4)	80 (60.2)	53 (39.8)	
Rarely (1–2 times a week)	38 (15.8)	18 (47.4)	20 (52.6)	
BMI				
Normal	141 (58.8)	77 (54.6)	64 (45.4)	9.918 (0.013∗)
Overweight	79 (32.9)	56 (70.9)	23 (29.1)	
Obesity	20 (8.3)	16 (80.0)	4 (20.0)	
Central obesity (waist‐to‐height ratio)				
< 0.49	106 (44.2)	56 (52.8)	50 (47.2)	8.619 (0.013∗)
0.5–0.59	116 (48.3)	78 (67.2)	38 (32.8)	
≥ 0.6	18 (7.5)	15 (83.3)	3 (16.7)	
Physical activity				
Low	162 (67.5)	96 (59.3)	66 (40.7)	2.735 (0.255)
Moderate	67 (27.9)	44 (65.7)	23 (34.3)	
High	11 (4.6)	9 (81.8)	2 (18.2)	
Average sitting time				
≤8 h	117 (48.8)	75 (64.1)	42 (35.9)	0.395 (0.529)
> 8 h	123 (51.3)	74 (60.2)	49 (39.8)	
Average sleep duration				
≤ 7 h	116 (48.3)	72 (62.1)	44 (37.9)	0.001 (0.996)
> 7 h	124 (51.7)	77 (62.12)	47 (37.9)	

^∗^Statistical significance at *p* < 0.05.

Health literacy scores varied across different domains, with participants demonstrating higher competency in “Navigating the healthcare system” (mean = 20.28 ± 3.80) and “Understanding health information well enough to know what to do” (mean = 18.30 ± 3.07). However, those with hypertension had significantly lower scores in key domains, including “Feeling understood and supported by healthcare providers” (*p* = 0.020), “Actively managing my health” (*p* = 0.008), “Social support for health” (*p* = 0.045), and “Ability to actively engage with healthcare providers” (*p* = 0.033). The overall health literacy score was also significantly lower among hypertensive individuals compared to nonhypertensive participants (134.86 ± 15.47 vs. 139.30 ± 13.84, *p* = 0.022). (Table 4).

**Table 4:** Health literacy scores across HLQ domains.

**HLQ domain**	**Mean score ± SD (95% CI for mean)**	**Hypertension**		***p* value**
		**Presence (mean ± SD)**	**Absence (mean ± SD)**	
Feeling understood and supported by healthcare providers (4 items)	10.21 ± 2.36 (9.90–10.49)	9.96 ± 2.62	10.63 ± 1.79	0.020∗
Having sufficient information to manage my health (4 items)	10.40 ± 1.84 (10.18–10.65)	10.34 ± 1.87	10.49 ± 1.80	0.549
Actively managing my health (5 items)	13.38 ± 2.29 (13.04–13.66)	13.04 ± 2.16	13.93 ± 2.41	0.008∗
Social support for health (5 items)	14.70 ± 2.27 (14.43–14.99)	14.47 ± 2.55	15.07 ± 1.66	0.045∗
Appraisal of health information (5 items);	14.37 ± 2.06 (14.09–14.63)	14.38 ± 2.15	14.36 ± 1.89	0.962
Ability to actively engage with healthcare providers (5 items);	17.38 ± 3.05 (17.01–17.76)	17.06 ± 3.30	17.90 ± 2.51	0.033∗
Navigating the healthcare system (6 items);	20.28 ± 3.80 (19.80–20.72)	19.87 ± 3.92	20.93 ± 3.50	0.028∗
Ability to find good health information (5 items);	17.53 ± 2.95 (17.19–17.92)	17.44 ± 3.10	17.68 ± 2.70	0.545
Understanding health information well enough to know what to do (5 items).	18.30 ± 3.07 (17.92–18.68)	18.28 ± 2.97	18.30 ± 3.23	0.963
Overall score (44 items)	136.55 ± 15.00 (134.53–138.48)	134.86 ± 15.47	139.30 ± 13.84	0.022∗

^∗^Statistical significance at *p* < 0.05.

The independent variables with a statistically significant relationship to hypertension in the bivariate analysis were included in the final model for multiple logistic regression analysis. A variance inflation factor (VIF) test was conducted to check for multicollinearity among the independent variables, with the highest reported VIF value being 1.891, confirming the absence of multicollinearity (VIF < 2.0). The adjusted logistic regression model accounted for key demographic, behavioral, and anthropometric variables. Males were found to have twice the odds of experiencing hypertension as compared to their female counterparts (aOR 2.237, 95% CI 1.170–4.276; *p* = 0.015). Alcohol consumption in the past 30 days was found to have fourfold increase in odds of hypertension (aOR 4.732, 95% CI 1.386–16.160; *p* = 0.013) compared to those who never consumed alcohol. In reference to those who never consume processed food, those who frequently consume processed foods (≥ 7 times per week) have twice the odds of hypertension (aOR 2.640, 95% CI 1.024–7.096; *p* = 0.046). Anthropometric factors remained significant predictors. Overweight participants had 1.8 times higher odds of hypertension (aOR 1.819, 95% CI 1.658–3.423; *p* = 0.025), and obese participants had 1.6 times higher odds (aOR 1.575, 95% CI 1.072–7.157; *p* = 0.043). Similarly, central obesity, measured as a waist‐to‐height ratio ≥ 0.6, increased the likelihood of hypertension by 1.967 times (aOR 1.967, 95% CI 1.162–7.843; *p* = 0.041) relative to participants with a ratio below 0.49 (Table 5).

**Table 5:** Logistic regression analysis of factors associated with hypertension.

**Variables**	**Hypertension**			
	**cOR (95% CI)**	***p* value**	**aOR (95% CI)**	***p* value**
Age				
20–30 years	**Ref**		**Ref**	
30–40 years	1.910 (1.089–3.352)	0.024	1.770 (0.822–3.811)	0.144
≥ 40 years	3.896 (1.247–12.168)	0.019	2.307 (0.552–9.646)	0.252
Gender				
Male	2.786 (1.611–4.818)	< 0.001	2.237 (1.170–4.276)	0.015∗
Female	**Ref**		**Ref**	
Duration in job				
< 1 years	**Ref**		**Ref**	
1–4 years	0.939 (0.460–1.917)	0.862	0.630 (0.281–1.415)	0.630
5–9 years	0.770 (0.374–1.587)	0.479	0.519 (0.212–1.274)	0.519
≥ 10 years	2.390 (1.022–5.589)	0.045∗	0.821 (0.248–2.718)	0.821
Alcohol consumption (past 30 days)				
Yes	3.233 (1.108–9.435)	0.032∗	4.732 (1.386–16.160)	0.013∗
No	1.753 (1.010–3.043)	0.046∗	1.154 (0.610–2.186)	0.058
Never consume alcohol	Ref		Ref	
Cooking oil in household				
Mustard oil	Ref		Ref	
Sunflower oil	1.819 (1.044–3.170)	0.035∗	1.728 (0.926–3.224)	0.086
Other oil (plant based)	3.405 (0.844–13.120)	0.075	2.985 (0.694–12.830)	0.142
Consumption of processed food				
Often (≥ 7 times a week)	3.148 (1.398–7.243)	0.007∗	2.640 (1.024–7.096)	0.046∗
Sometimes (3–6 times a week)	1.677 (0.812–3.464)	0.162	1.482 (0.614–3.575)	0.381
Rarely (1–2 times a week)	Ref		Ref	
BMI				
Normal	Ref		Ref	
Overweight	2.024 (1.124–3.643)	0.019∗	1.819 (1.658–3.423)	0.025∗
Obesity	3.325 (1.158–10.445)	0.040∗	1.575 (1.072–7.157)	0.043∗
Central obesity (waist‐to‐height ratio)				
< 0.49	Ref		Ref	
0.5–0.59	1.833 (1.129–3.157)	0.029∗	1.289 (1.062–2.665)	0.035∗
≥ 0.6	4.645 (1.200–16.330)	0.024∗	1.967 (1.162–7.843)	0.041∗

^∗^Statistical significance at *p* < 0.05.

## 4. Discussion

This study assessed the prevalence and determinants of hypertension among commercial bank employees in Sunsari, Nepal, revealing a high burden of hypertension within this occupational group. The findings indicate that 62.1% of participants were hypertensive, which is significantly higher than the 24.5% national prevalence reported in the WHO STEPS 2019 survey and the 28.4% (95% CI 22.4–34.7%) pooled prevalence reported in a systematic review of hypertension studies in Nepal [10, 20, 24]. These findings underscore that hypertension among bank workers is substantially higher than in the general adult population in Nepal. The observed higher prevalence of hypertension among bank workers aligns with findings from neighboring countries, where hypertension among bank employees has been reported at 24.44% in Bangladesh [15], 44.3%–69.5% among bank employees India [14, 16]. The elevated prevalence of hypertension among bank employees can be attributed to occupational stress, prolonged sedentary behavior, and unhealthy lifestyle practices [13, 19].

Despite the high prevalence, awareness of hypertension remains alarmingly low. In this study, only 7.5% of hypertensive respondents were aware of their blood pressure status. This is in line with another study from Nepal where more than half of the hypertensive patients were unaware of their condition [9, 25–28]. The WHO has acknowledged hypertension as a “silent killer,” emphasizing its rapid increase in low‐ and middle‐income countries and its contribution to cardiovascular diseases [29]. This lack of awareness underscores the urgent need for public health interventions promoting routine blood pressure monitoring, early detection, and lifestyle modifications to prevent severe health complications. Nepal has acknowledged this rising concern of hypertension in its policy documents, such as the National Health Policy‐2019 and Nepal Health Sector Strategic Plan 2023‐2030, reflecting the need to address the wider determinants of health by making citizens responsible for their health [30, 31]. However, given the disproportionately high burden among bank employees, targeted workplace interventions are essential. These many include stress management programs, routine health screenings, and awareness campaigns are necessary to mitigate hypertension risks in this occupational group. In addition, workplace‐based improvements, such as offering healthy cafeteria options and discouraging the availability of processed foods, along with initiatives to promote physical activity such as short active breaks, subsidized gym memberships, or workplace exercise sessions could be integrated into comprehensive workplace health strategies. These measures are particularly important given the high prevalence of obesity among bank employees and its strong link to hypertension. The bivariate analysis revealed that individuals aged ≥ 40 years had three times the odds of developing hypertension, while those aged 30–39 years had nearly twice the odds. This finding is consistent with previous research conducted in low‐ and middle‐income countries, as well as in member states of the South Asian Association for Regional Cooperation (SAARC) [32, 33]. However, after adjusting for other factors in the multivariate model, the association between age and hypertension was diminished, suggesting that additional lifestyle and occupational factors may play a more significant role in hypertension. Furthermore, gender‐based differences were also observed, with males exhibiting twice the odds of hypertension compared to females (aOR 2.237, 95% CI 1.170–4.276). This align with another study from Nepal focusing on hypertension among mid‐age population where males had nearly twice the risk of hypertension (aOR 1.903, 95% CI 1.184–3.030) [28]. Additionally, the WHO STEPS survey (2019) found that hypertension prevalence in Nepal was significantly higher in men than in women (29.8% vs. 19.7%) [11]. These findings emphasize the importance of workplace strategies that address hypertension risk factors among high‐risk groups, particularly men and older employees.

Behavioral risk factors, particularly, alcohol consumption and dietary habits, were significantly associated with hypertension. Participants who consumed alcohol in the past 30 days had nearly fivefold increased odds of hypertension compared to non‐drinkers (aOR 4.732, 95% CI 1.386–16.160), underscoring the hypertensive effects of alcohol consumption. A similar trend was observed in a recent study among middle‐aged individuals in rural Nepal, where alcohol consumers had twice the odds of being hypertensive [28]. The positive association between alcohol consumption and hypertension has been reported in various countries [5, 32, 34]. A meta‐analysis of 15 randomized controlled trials further confirmed a dose–response relationship between alcohol reduction and mean blood pressure reduction [35]. These findings support the need for workplace‐based alcohol awareness campaigns and counseling services for employees.

Frequent consumption of processed foods emerged as a significant determinant, with individuals consuming processed foods ≥7 times per week having more than double the odds of hypertension (aOR: 2.61, 95% CI: 1.024–7.096). Previous studies have shown a strong correlation between processed food consumption and hypertension, particularly in urban populations where dietary habits have shifted toward high‐sodium and high‐fat foods [36, 37]. White‐collar workers, including corporate employees and bank workers, are at a heightened risk of consuming processed foods due to job‐related constraints such as long working hours, limited access to home‐cooked meals, and the convenience of fast food options [38, 39]. Literature suggest that consumption of processed foods high in salt is increasing in developing nations resulting in the rise of several chronic conditions such as obesity, diabetes, heart disease and stroke, cancers, and so on [40, 41]. Given these trends, these findings emphasize the need for targeted nutritional interventions in the workplace, such as replacing processed foods with healthier alternatives and creating awareness campaigns to promote healthy eating.

Anthropometric factors, particularly BMI and central obesity, remained strong predictors of hypertension. Overweight participants had 1.8 times higher odds (aOR 1.819, 95% CI 1.658–3.423; *p* = 0.025), while those experiencing obesity were 1.5 times more at odds (aOR 1.575, 95% CI 1.072–7.157; *p* = 0.043) of being hypertensive. Central obesity, defined as a waist‐to‐height ratio ≥ 0.6, was associated with nearly a twofold increase in hypertension risk (aOR 1.967, 95% CI 1.162–7.843; *p* = 0.041). These findings align with studies conducted in Nepal and other South Asian nations, reinforcing the strong link between obesity and hypertension [28, 33]. Obesity has established itself as a rising public health problem linked with several chronic conditions throughout the world [32, 33]. It has been linked with hypertension in countries of different economies such as Ethiopia [42], South Africa [43], Saudi Arabia [44], and China [45]. The impact of obesity on hypertension is well‐documented regardless of economic context [5, 32]. According to the American College of Cardiology/American Heart Association, a clear dose–response relationship exists between BMI and blood pressure, making BMI a crucial modifiable risk factor for hypertension [46]. Workplace wellness programs promoting physical activity, balanced diets, and regular monitoring of BMI and waist circumference are vital to address this modifiable risk factor. To facilitate this local governments can also play a role by encouraging regular physical activity and healthy eating to prevent obesity and related hypertension.

Health literacy, as measured across nine domains of the Health Literacy Questionnaire (HLQ), exhibited varying levels of competency among participants. While some domains demonstrated relatively high competency, significant gaps were observed in key areas that may impact hypertension awareness, prevention, and management. Hypertensive individuals had significantly lower scores in domains such as “Feeling understood and supported by healthcare providers,” “Actively managing my health,” “Social support for health,” “Ability to actively engage with healthcare providers,” and “Navigating the healthcare system,” as well as a lower overall health literacy score. These findings suggest that inadequate health literacy may contribute to poor hypertension awareness, delayed diagnosis, and ineffective management, consistent with previous research linking low health literacy to chronic disease burden [17, 18], including hypertension. Similar patterns have been observed in other occupational settings, emphasizing the role of health literacy in disease prevention and management [19, 47]. Given the high burden of hypertension in this population and poor health literacy, workplace‐based interventions, including health literacy programs, targeted health education, and routine screenings, could enhance engagement with healthcare services and improve hypertension prevention and control strategies.

The findings of this study underscore the urgent need for workplace‐based hypertension prevention programs tailored to the needs of white‐collar workers. Given the high prevalence of hypertension and its association with modifiable risk factors, targeted interventions such as workplace stress management programs, physical activity promotion, and nutrition education should be prioritized. This study provides valuable insights into the prevalence and determinants of hypertension among commercial bank employees in Sunsari, Nepal, using random sampling, validated tools, and standardized hypertension classification criteria to enhance methodological rigor.

Despite these strengths there are some limitations which needs to be acknowledged. The study was conducted in a single district, which may limit generalizability to other occupational settings in Nepal, though the use of random sampling within diverse bank locations helps improve representativeness. Additionally, even the efforts of ensuring anonymity and private data collection were made to mitigate the introduction of risk of recall and social desirability bias due to the reliance on self‐reported behavioral data, particularly for alcohol and tobacco use, yet some misreporting may persist. Furthermore, due to time constraints and the length of the questionnaire, some variables related to occupational settings, such as job‐related stress, work hours, and shift patterns, which could influence hypertension risk, were not explored. These factors are highly relevant to hypertension risk and future research could incorporate such occupational variables to provide a more comprehensive understanding of work‐related hypertension risk factors. Additionally, multi‐site studies covering different geographical regions and industries could enhance the understanding about hypertension in occupational setup.

## 5. Conclusion

This study revealed a high prevalence of hypertension among commercial bank employees in Sunsari, Nepal, exceeding national estimates. Male gender, alcohol consumption, frequent processed food intake, and obesity were identified as significant risk factors, emphasizing the need for targeted lifestyle interventions. Additionally, low health literacy particularly, in domains related to feeling understood and supported by healthcare providers, self‐care management, social support, and navigating the healthcare system, was significantly associated with hypertension, indicating barriers to effective awareness and control. Given the low diagnosis practice and observed health literacy gaps, workplace‐based interventions including routine screenings, targeted health education, and initiatives to enhance health literacy and self‐management skills are essential to improve hypertension prevention and management within this occupational group.

## Conflicts of Interest

The authors declare no conflicts of interest.

## Author Contributions

**S.K.**: conceptualization, investigation, data curation, project administration, validation, methodology, data analysis, and writing—original draft. **S.S.B.**: conceptualization, supervision, methodology, and writing—review and editing. **S.P.**: visualization, data analysis, writing—original draft, and writing—review and editing. **D.K.**: data analysis, writing—original draft, and writing—review and editing. **A.G.**: supervision, and writing—review and editing. **D.K.Y.**: supervision, and writing—review and editing. **P.K.P.**: conceptualization, supervision, and writing—review and editing.

## Funding

No funding was received for this manuscript.

## Data Availability

The data that support the findings of this study are available from the corresponding author upon reasonable request.
